# Supplementary material for: Stability of Estimated Premorbid Cognitive Ability over Time after Minor Stroke and Its Relationship with Post-Stroke Cognitive Ability
Source: Brain Sci. 2019 May 22;9(5):117. doi: 10.3390/brainsci9050117 (PMC6562568; doi:10.3390/brainsci9050117)
Supplement: Supplementary file 1 [file brainsci-09-00117-s001.pdf]

Figure S1. Residual plots for linear mixed models examining the associations between time, demographic and vascular risk factors with NART IQ and ACE-R scores by time-point

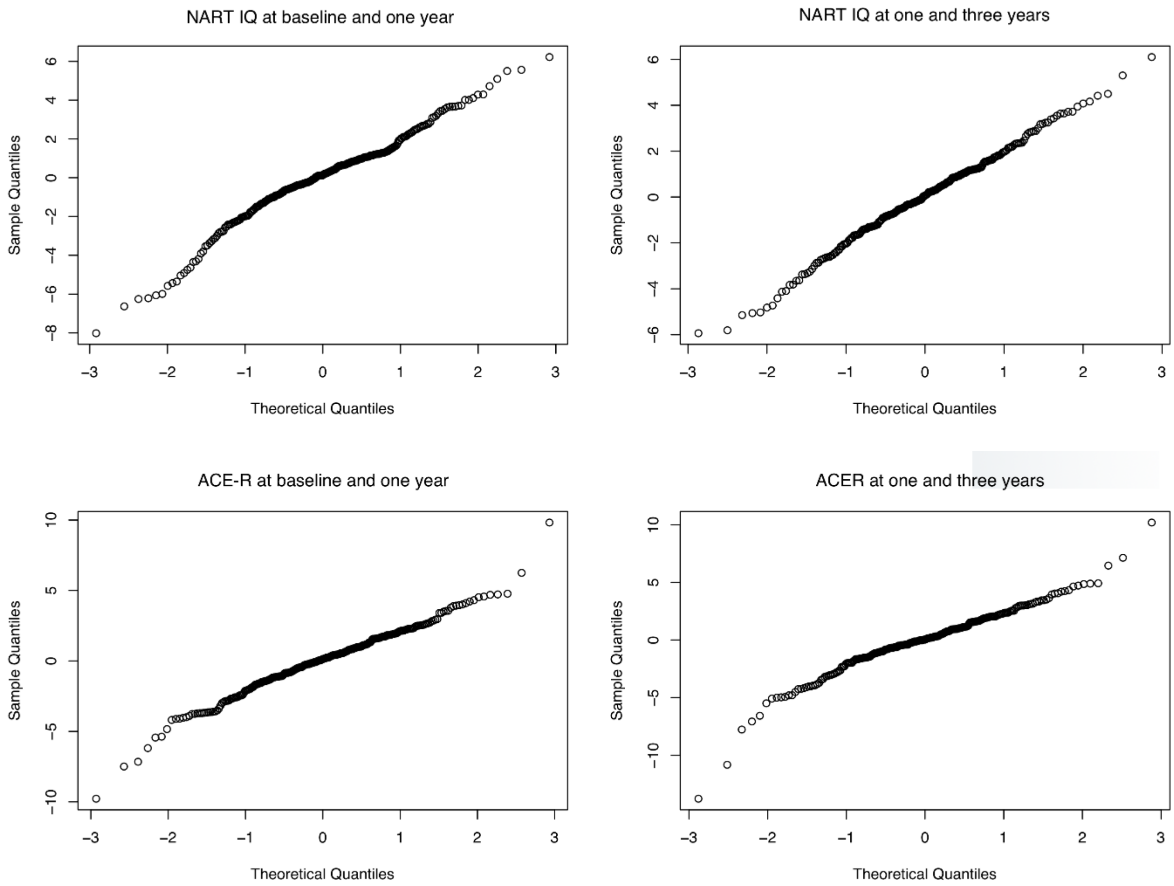

Table S1. NART IQ and ACE-R scores at and between each time-point for all patients with data available at all time-points.

	Scores				Difference scores		
	**1-3 months**	**1 year**	**3 years**		**1-3 months to 1 year**	**1 to 3 years**	**1-3 months to 3 years**
				**NART IQ (n=90)**			
**Mean±SD (range)**	116.35±8.32 (94.66-127.70)	117.34±9.25 (9.30-127.70)	113.26±8.78 (92.18-127.70)	**Mean change (95% CI)**	0.991 (0.07 to 1.91)*	-4.075 (-4.98 to -3.17)**	-3.084 (-3.92 to -2.25)**
				**Decreased n(%)**	27 (30.34%)	72 (79.12%)	72 (77.42%)
				**Increased n(%)**	51 (57.30%)	12 (13.19%)	14 (15.05%)
				**No change n(%)**	11 (12.36%)	7 (7.69%)	7 (7.53%)
				**ACE-R (n=96)**			
**Mean±SD (range)**	89.14±7.70 (69.00-100.00)	90.00±7.37 (71.00-100.00)	88.86±9.07 (54.00-100.00)	**Mean change (95% CI)**	0.865 (0.07 to 1.80)	-1.135 (-2.27 to -0.00)*	0.271 (-1.48 to 0.94)
**No impairment n(%)[a]**	59 (61.46%)	64 (66.67%)	59 (61.46%)	**Decreased n(%)**	36 (37.50%)	40 (41.67%)	53 (55.21%)
**Mild impairment n(%)[b]**	15 (15.63%)	16 (16.67%)	18 (18.75%)	**Increased n(%)**	47 (48.96%)	45 (46.88%)	38 (39.58%)
**Severe impairment n(%)[c]**	22 (22.92%)	16 (16.67%)	19 (19.79%)	**No change n(%)**	13 (13.54%)	11 (11.46%)	5 (5.21%)

* $p<0.05$

** $p<0.001$

[a] ACE-R scores ≥89

[b] ACE-R scores 83-88

[c] ACE-R scores ≤ 82

Table S2. Linear mixed effects model showing the associations between time and NART IQ and ACE-R adjusted for baseline characteristics for patients with data available at all time-points.

	**Changes in scores between 1 month and 3 years**			**Changes in scores between 1 and 3 years**		
	**B**	**95% CI**	***p***	**B**	**95% CI**	***p***
			**NART IQ**			
***Time** (years)*	*0.975*	*(0.051, 1.898)*	*0.038*	*-4.111*	*(-5.019, -3.204)*	*<0.001*
**Age**	0.109	(-0.083, 0.301)	0.287	0.118	(-0.078, 0.315)	0.258
**Sex** (male)	1.699	(-1.525, 4.923)	0.322	1.650	(-1.643, 4.943)	0.345
**Baseline Fazekas**	0.242	(-1.016, 1.500)	0.717	0.071	(-1.214, 1.356)	0.917
**Stroke subtype** (lacunar)	1.656	(-1.656, 4.968)	0.348	1.586	(-1.797, 4.969)	0.379
**NIHSS**	-0.537	(-1.861, 0.786)	0.446	-0.414	(-1.767, 0.938)	0.565
**Hypertension** (yes)	2.608	(-1.121, 6.336)	0.189	2.696	(-1.112, 6.504)	0.184
**Smoker** (yes)	0.169	(-3.724, 4.062)	0.935	-0.328	(-4.304, 3.648)	0.877
**Years of education**	1.192	(0.639, 1.745)	<0.001	1.265	(0.700, 1.830)	<0.001
			**ACE-R**			
***Time** (years)*	*0.872*	*(-0.050, 1.795)*	*0.063*	*-1.160*	*(-2.296, -0.023)*	*0.045*
**Age**	-0.057	(-0.213, 0.099)	0.492	-0.145	(-0.310, 0.020)	0.097
**Sex** (male)	-0.017	(-2.602, 2.568)	0.990	-0.585	(-3.320, 2.149)	0.687
**Baseline Fazekas**	-0.277	(-1.301, 0.748)	0.611	-0.293	(-1.377, 0.790)	0.610
**Stroke subtype** (lacunar)	0.510	(-2.179, 3.199)	0.721	1.010	(-1.834, 3.854)	0.504
**NIHSS**	-0.686	(-1.770, 0.398)	0.233	-0.553	(-1.700, 0.594)	0.364
**Hypertension** (yes)	3.213	(0.245, 6.181)	0.042	4.055	(0.916, 7.195)	0.015
**Smoker** (yes)	1.447	(-1.695, 4.590)	0.386	1.029	(-2.295, 4.353)	0.560
**Years of education**	0.993	(0.546, 1.439)	<0.001	1.061	(0.589, 1.533)	<0.001

Table S3. Full linear mixed effects models showing factors associated with changes in NART IQ at each time-point

	B	95% CI	*p*	B	95% CI	*p*	B	95% CI	*p*	B	95% CI	*p*	B	95% CI	*p*	B	95% CI	*p*	B	95% CI	*p*	B	95% CI	*p*
**1-3 months and 1 year**																								
**NART IQ**																								
**Time**	1.339	(-3.545, 6.223)	0.588	1.878	(0.582, 3.173)	0.005	2.954	(1.306, 4.602)	0.001	1.122	(0.035, 2.210)	0.043	1.163	(0.066, 2.260)	0.038	1.695	(0.121, 3.269)	0.035	1.672	(0.692, 2.651)	0.001	4.277	(1.060, 7.494)	0.010
**Age**	0.095	(-0.038, 0.229)	0.160	0.096	(-0.034, 0.225)	0.146	0.095	(-0.035, 0.224)	0.150	0.097	(-0.033, 0.226)	0.142	0.096	(-0.033, 0.226)	0.144	0.096	(-0.033, 0.226)	0.144	0.096	(-0.034, 0.225)	0.146	0.099	(-0.031, 0.228)	0.134
**Sex** (male)	1.237	(-1.198, 3.673)	0.316	1.546	(-0.998, 4.090)	0.231	1.220	(-1.214,3.654)	0.323	1.248	(-1.186, 3.682)	0.312	1.248	(-1.186, 3.682)	0.312	1.248	(-1.187, 3.684)	0.312	1.249	(-1.185, 3.684)	0.312	1.235	(-1.195, 3.664)	0.316
**Baseline Fazekas**	0.178	(-0.656, 1.011)	0.674	0.176	(-0.657, 1.010)	0.676	0.402	(-0.458, 1.262)	0.357	0.169	(-0.664, 1.002)	0.688	0.175	(-0.657, 1.008)	0.677	0.176	(-0.657, 1.009)	0.676	0.185	(-0.648, 1.018)	0.661	0.176	(-0.655, 1.008)	0.675
**Stroke subtype** (lacunar)	1.033	(-1.499, 3.564)	0.421	1.024	(-1.508, 3.555)	0.425	1.082	(-1.448, 3.612)	0.399	0.704	(-1.926, 3.334)	0.597	1.027	(-1.502, 3.557)	0.423	1.033	(-1.497, 3.564)	0.420	1.021	(-1.509, 3.552)	0.426	1.018	(-1.507, 3.544)	0.426
**NIHSS**	-0.213	(-1.342, 0.916)	0.709	-0.219	(-1.348, 0.910)	0.702	-0.211	(-1.340, 0.917)	0.712	-0.218	(-1.347, 0.910)	0.702	-0.340	(-1.513, 0.833)	0.567	-0.214	(-1.342, 0.915)	0.709	-0.212	(-1.341, 0.917)	0.711	-0.220	(-1.347, 0.906)	0.699
**Hypertension** (yes)	2.821	(0.139, 5.504)	0.039	2.855	(0.172, 5.538)	0.037	2.811	(0.131, 5.492)	0.040	2.828	(0.146, 5.509)	0.039	2.824	(0.143, 5.504)	0.039	2.970	(0.162, 5.778)	0.038	2.834	(0.152, 5.515)	0.039	2.815	(0.139, 5.491)	0.039
**Smoker** (yes)	-0.566	(-3.275, 2.143)	0.680	-0.552	(-3.261, 2.157)	0.687	-0.512	(-3.220, 2.196)	0.709	-0.554	(-3.261, 2.154)	0.686	-0.566	(-3.273, 2.141)	0.679	-0.560	(-3.268,2.148)	0.683	-0.256	(-3.073, 2.562)	0.858	-0.573	(-3.276, 2.129)	0.675
**Years of education**	1.284	(0.860, 1.708)	<0.001	1.285	(0.861, 1.709)	<0.001	1.289	(0.865, 1.713)	<0.001	1.287	(0.863, 1.711)	<0.001	1.286	(0.862, 1.710)	0.000	1.284	(0.860, 1.708)	<0.001	1.281	(0.857, 1.706)	<0.001	1.400	(0.958, 1.842)	<0.001
**Variable + Interactions**																								
**Age:Time**	0.097	(-0.039, 0.233)	0.160																					
**Sex:Time**				0.847	(-1.760, 3.453)	0.521																		
**Baseline Fazekas:Time**							-0.123	(-1.004, 0.758)	0.782															
**Stroke subtype:Time**										1.454	(-1.238, 4.145)	0.287												
**NIHSS:Time**													-0.062	(-1.253, 1.130)	0.918									
**Hypertension:Time**																2.642	(-0.222, 5.506)	0.070						
**Smoker:Time**																			-0.957	(-3.836, 1.923)	0.512			
**Years of education:Time**																						1.165	(0.721, 1.608)	<0.001
**1-3 years**																								
**Time**	-2.107	(-4.706, 0.492)	0.111	-2.327	(-2.997, -1.657)	<0.001	-2.633	(-3.551, -1.716)	<0.001	-1.723	(-2.283, -1.164)	<0.001	-2.129	(-2.727, -1.531)	<0.001	-2.36	(-3.215, -1.505)	<0.001	-2.152	(-2.658, -1.646)	<0.001	-4.332	(-6.003, -2.661)	<0.001
**Age**	0.098	(-0.061, 0.257)	0.223	0.095	(-0.048, 0.238)	0.191	0.094	(-0.049, 0.237)	0.193	0.095	(-0.048, 0.239)	0.191	0.095	(-0.048, 0.238)	0.188	0.095	(-0.048, 0.238)	0.191	0.094	(-0.049, 0.237)	0.193	0.101	(-0.042, 0.243)	0.165
**Sex** (male)	1.504	(-1.098, 4.107)	0.254	1.214	(-1.866, 4.293)	0.436	1.502	(-1.100, 4.104)	0.255	1.531	(-1.081, 4.142)	0.248	1.509	(-1.093, 4.110)	0.252	1.501	(-1.101, 4.103)	0.255	1.496	(-1.106, 4.098)	0.257	1.502	(-1.096, 4.099)	0.254
**Baseline Fazekas**	0.010	(-0.892, 0.913)	0.982	0.010	(-0.892, 0.912)	0.983	-0.242	(-1.281, 0.797)	0.644	-0.030	(-0.936, 0.876)	0.947	0.006	(-0.896, 0.909)	0.989	0.009	(-0.893, 0.912)	0.984	0.001	(-0.907, 0.904)	0.998	-0.023	(-0.924, 0.878)	0.961
**Stroke subtype** (lacunar)	0.875	(-1.846, 3.596)	0.525	0.877	(-1.842, 3.596)	0.524	0.931	(-1.791, 3.653)	0.499	2.980	(-0.160, 6.119)	0.063	0.880	(-1.839, 3.599)	0.522	0.886	(-1.834, 3.607)	0.519	0.882	(-1.838, 3.601)	0.521	0.846	(-1.869, 3.561)	0.538
**NIHSS**	0.034	(-1.163, 1.232)	0.955	0.032	(-1.165, 1.229)	0.958	0.028	(-1.169, 1.226)	0.963	0.035	(-1.167, 1.237)	0.954	0.213	(-1.166, 1.592)	0.760	0.031	(-1.166, 1.229)	0.959	0.037	(-1.161, 1.234)	0.952	0.041	(-1.155, 1.236)	0.946
**Hypertension** (yes)	2.311	(-0.571, 5.192)	0.115	2.311	(-0.569, 5.191)	0.115	2.309	(-0.573, 5.190)	0.115	2.283	(-0.609, 5.174)	0.120	2.315	(-0.565, 5.195)	0.114	2.012	(-1.383, 5.406)	0.242	2.302	(-0.579, 5.183)	0.116	2.343	(-0.533, 5.218)	0.109
**Smoker** (yes)	-1.610	(-4.546, 1.326)	0.279	-1.599	(-4.533, 1.335)	0.282	-1.559	(-4.495, 1.378)	0.295	-1.596	(-4.540, 1.349)	0.285	-1.598	(-4.532, 1.336)	0.282	-1.598	(-4.534, 1.337)	0.282	-1.051	(-4.446, 2.344)	0.540	-1.584	(-4.513, 1.345)	0.286
**Years of education**	1.190	(0.754, 1.625)	<0.001	1.191	(0.756, 1.626)	<0.001	1.189	(0.754, 1.624)	<0.001	1.197	(0.760, 1.634)	<0.001	1.191	(0.756, 1.626)	<0.001	1.191	(0.756, 1.627)	<0.001	1.190	(0.755, 1.625)	<0.001	0.873	(0.375, 1.372)	0.001
**Variable + Interactions**																								
**Age:Time**	0.0963	(-0.050, 0.243)	0.194																					
**Sex:Time**				1.3677	(-1.345, 4.081)	0.319																		
**Baseline Fazekas:Time**							-0.103	(-1.035, 0.828)	0.826															
**Stroke subtype:Time**										1.842	(-0.979, 4.663)	0.198												
**NIHSS:Time**													0.116	(-1.122, 1.354)	0.852									
**Hypertension:Time**																2.176	(-0.815, 5.168)	0.152						
**Smoker:Time**																			-1.369	(-4.393, 1.656)	0.371			
**Years of education:Time**																						1.046	(0.598, 1.495)	<0.001

Table S4. Full linear mixed effects models showing factors associated with changes in current ACE-R scores at each time-point.

	1-3 months and 1 year																										
	B	95% CI	*p*	B	95% CI	*p*	B	95% CI	*p*	B	95% CI	*p*	B	95% CI	*p*	B	95% CI	*p*	B	95% CI	*p*	B	95% CI	*p*	B	95% CI	*p*
	ACE-R																										
**Time**	-2.191	(-7.337, 2.954)	0.401	-0.001	(-1.389, 1.387)	0.999	0.207	(1.597, 2.011)	0.820	-0.020	(-1.177, 1.137)	0.973	-0.536	(-1.683, 0.612)	0.357	0.073	(-1.601, 1.746)	0.932	0.595	(-0.452, 1.642)	0.263	-6.217	(-18.303, 5.869)	0.310	-2.157	(-5.616, 1.302)	0.219
**Age**	-0.239	(-0.345, -0.133)	<0.001	-0.223	(-0.323, -0.122)	<0.001	-0.223	(-0.323, -0.122)	<0.001	-0.222	(-0.323, -0.122)	0.000	-0.222	(-0.323, -0.122)	<0.011	-0.223	(-0.323, -0.122)	<0.001	-0.223	(-0.324, -0.122)	0.000	-0.224	(-0.324, -0.123)	0.000	-0.225	(-0.326, -0.125)	<0.001
**Sex** (male)	-0.202	(-2.085, 1.681)	0.832	-0.370	(-2.417, 1.677)	0.721	-0.216	(-2.104, 1.671)	0.821	-0.207	(-2.097, 1.683)	0.829	-0.176	(-2.068, 1.715)	0.854	-0.222	(-2.111, 1.666)	0.816	-0.187	(-2.078, 1.703)	0.845	-0.218	(-2.105, 1.669)	0.819	-0.222	(-2.107, 1.663)	0.816
**Baseline Fazekas**	-0.214	(-0.862, 0.435)	0.515	-0.221	(-0.870, 0.428)	0.502	-0.221	(-0.915, 0.473)	0.529	-0.225	(-0.876, 0.425)	0.494	-0.224	(-0.875, 0.427)	0.498	-0.221	(-0.871, 0.429)	0.502	-0.208	(-0.859, 0.443)	0.528	-0.202	(-0.853, 0.448)	0.539	-0.222	(-0.871, 0.427)	0.500
**Stroke subtype** (lacunar)	-0.107	(-2.061, 1.847)	0.914	-0.097	(-2.053, 1.860)	0.922	-0.099	(-2.058, 1.861)	0.921	-0.327	(-2.433, 1778)	0.759	-0.105	(-2.068, 1.858)	0.916	-0.099	(-2.058, 1.860)	0.921	-0.112	(-2.074, 1.850)	0.910	-0.113	(-2.072, 1.845)	0.909	-0.094	(-2.050, 1.862)	0.925
**NIHSS**	-0.277	(-1.141, 0.587)	0.527	-0.272	(-1.138, 0.593)	0.534	-0.275	(-1.142, 0.591)	0.530	-0.279	(-1.146, 0.588)	0.525	-0.609	(-1.541, 0.323)	0.198	-0.275	(-1.141, 0.591)	0.530	-0.275	(-1.142, 0.593)	0.532	-0.280	(-1.146, 0.586)	0.523	-0.268	(-1.133, 0.597)	0.541
**Hypertension** (yes)	2.845	(0.762, 4.928)	0.008	2.826	(0.738, 4.915)	0.008	2.844	(0.755, 4.933)	0.008	2.856	(0.765, 4.947)	0.008	2.875	(0.783, 4.968)	0.007	2.761	(0.499, 5.033)	0.018	2.879	(0.787, 4.972)	0.007	2.878	(0.789, 4.967)	0.007	2.833	(0.747, 4.919)	0.008
**Smoker** (yes)	-0.159	(-2.243, 1.924)	0.880	-0.156	(-2.243, 1.930)	0.882	-0.151	(-2.240, 1.938)	0.887	-0.147	(-2.238, 1.944)	0.890	-0.164	(-2.257, 1.929)	0.877	-0.154	(-2.242, 1.935)	0.885	0.388	(-1.859, 2.635)	0.733	-0.141	(-2.229, 1.948)	0.894	-0.143	(-2.229, 1.943)	0.892
**NART**	0.320	(0.215, 0.425)	<0.001	0.321	(0.215, 0.426)	<0.001	0.319	(0.214, 0.426)	<0.001	0.317	(0.212, 0.423)	0.000	0.311	(0.206, 0.416)	<0.011	0.320	(0.214, 0.425)	<0.001	0.315	(0.209, 0.420)	0.000	0.290	(0.171, 0.408)	0.000	0.326	(0.220, 0.431)	<0.001
**Years of education**	0.506	(0.155, 0.856)	0.005	0.509	(0.158, 0.860)	0.005	0.511	(0.159, 0.863)	0.005	0.516	(0.164, 0.868)	0.004	0.526	(0.174, 0.878)	0.004	0.511	(-0.159, 0.862)	0.005	0.512	(0.160, 0.864)	0.005	0.519	(0.168, 0.871)	0.004	0.407	(0.026, 0.788)	0.037
**Variable + Interactions**																											
**Age:Time**	-0.202	(-0.311, -0.093)	<0.001																								
**Sex:Time**				-0.030	(-2.150, 2.090)	0.977																					
**Baseline Fazekas:Time**							-0.222	(-0.937, 0.494)	0.541																		
**Stroke subtype:Time**										0.191	(-1.995, 2.377)	0.863															
**NIHSS:Time**													0.115	(-0.840, 1.070)	0.812												
**Hypertension:Time**																2.943	(0.605, 5.280)	0.014									
**Smoker:Time**																			-0.835	(-3.165, 1.494)	0.479						
**NART:Time**																						0.345	(0.229, 0.461)	<0.001			
**Years of education:Time**																									0.603	(0.229, 0.977)	0.002
	1-3 years																										
**Time**	5.771	(2.560, 8.981)	0.001	0.803	(-0.114, 1.720)	0.085	1.250	(0.018, 2.482)	0.047	<0.001	(-0.783, 0.783)	1.000	0.683	(-0.138, 1.504)	0.102	0.444	(-0.709, 1.598)	0.446	0.089	(-0.619, 0.796)	0.804	-3.704	(-11.427, 4.020)	0.343	1.019	(-1.312, 3.350)	0.387
**Age**	-0.054	(-0.196, 0.087)	0.447	-0.204	(-0.315, -0.093)	0.000	-0.203	(-0.314, -0.092)	0.000	-0.205	(-0.315, -0.094)	0.000	-0.202	(-0.313, -0.091)	0.000	-0.203	(-0.314, -0.093)	<0.001	-0.201	(-0.312, -0.091)	<0.001	-0.203	(-0.313, -0.093)	<0.001	-0.205	(-0.316, -0.095)	<0.001
**Sex** (male)	-0.136	(-2.160, 1.888)	0.894	1.016	(-1,928, 3.960)	0.495	-0.202	(-2.209, 1.805)	0.842	-0.230	(-2.237, 1.776)	0.820	-0.192	(-2.199, 1.815)	0.850	-0.205	(-2.212, 1.802)	0.839	-0.167	(-2.175, 1.841)	0.869	-0.232	(-2.230, 1.765)	0.818	-0.210	(-2.215, 1.795)	0.836
**Baseline Fazekas**	-0.609	(-1.312, 0.094)	0.089	-0.639	(-1.340, 0.061)	0.073	-0.123	(-1.088, 0.842)	0.801	-0.610	(-1.308, 0.088)	0.086	-0.647	(-1.345, 0.051)	0.069	-0.641	(-1.338, 0.057)	0.071	-0.615	(-1.313, 0.083)	0.084	-0.653	(-1.348, 0.041)	0.065	-0.633	(-1.330, 0.065)	0.075
**Stroke subtype** (lacunar)	0.776	(-1.336, 2.889)	0.467	0.916	(-1.186, 3.018)	0.389	0.818	(-1.278, 2.914)	0.440	-1.008	(-3.990, 1.974)	0.504	0.912	(-1.181, 3.005)	0.389	0.910	(-1.184, 3.004)	0.390	0.919	(-1.174, 3.013)	0.385	0.913	(-1.169, 2.996)	0.386	0.915	(-1.176, 3.006)	0.387
**NIHSS**	-0.177	(-1.097, 0.744)	0.704	-0.203	(-1.118, 0.712)	0.660	-0.198	(-1.109, 0.713)	0.667	-0.207	(-1.118, 0.703)	0.652	0.227	(-1.063, 1.517)	0.727	-0.209	(-1.120, 0.702)	0.650	-0.218	(-1.129, 0.694)	0.636	-0.205	(-1.111, 0.701)	0.654	-0.210	(-1.120, 0.700)	0.648
**Hypertension** (yes)	2.404	(0.153, 4.654)	0.037	2.356	(0.114, 1.720)	0.040	2.359	(0.127, 4.591)	0.039	2.368	(0.136, 4.599)	0.038	2.373	(0.140, 4.605)	0.037	2.378	(-0.875, 5.632)	0.150	2.398	(0.165, 4.631)	0.036	2.356	(0.135, 4.577)	0.038	2.343	(0.111, 4.574)	0.040
**Smoker** (yes)	0.553	(-1.745, 2.852)	0.634	0.697	(-1.593, 2.988)	0.547	0.654	(-1.628, 2.937)	0.571	0.745	(-1.534, 3.025)	0.518	0.746	(-1.535, 3.027)	0.518	0.737	(-1.545, 3.019)	0.523	-1.491	(-4.670, 1.689)	0.354	0.749	(-1.521, 3.020)	0.514	0.738	(-1.541, 3.017)	0.522
**NART**	0.430	(0.312, 0.548)	<0.001	0.443	(0.324, 0.562)	0.000	0.446	(0.327, 0.564)	0.000	0.451	(0.333, 0.570)	<0.001	0.442	(0.323, 0.560)	0.000	0.444	(0.326, 0.563)	<0.001	0.442	(0.324, 0.561)	<0.001	0.380	(0.211, 0.549)	<0.001	0.448	(0.329, 0.567)	<0.001
**Years of education**	0.455	(0.090, 0.820)	0.015	0.464	(0.100, 0.828)	0.013	0.465	(0.103, 0.828)	0.012	0.450	(0.088, 0.813)	0.015	0.469	(0.106, 0.831)	0.012	0.465	(0.102, 0.827)	0.013	0.468	(0.106, 0.831)	0.012	0.456	(0.096, 0.817)	0.014	0.548	(0.064, 1.032)	0.027
**Variable + Interactions**																											
**Age:Time**	-0.137	(-0.255, -0.019)	0.024																								
**Sex:Time**				0.370	(-1.882, 2.622)	0.745																					
**Baseline Fazekas:Time**							-0.407	(-1.167, 0.353)	0.291																		
**Stroke subtype:Time**										0.022	(-2.291 ,2.335)	0.985															
**NIHSS:Time**													-0.005	(-1.011, 1.000)	0.992												
**Hypertension:Time**																2.367	(-0.108, 4.843)	0.061									
**Smoker:Time**																			-0.226	(-2.697, 2.245)	0.856						
**NART:Time**																						0.416	(0.286, 0.546)	<0.001			
**Years of education:Time**																									0.501	(0.113, 0.888)	0.012
